# Genomics reveals the novel species placement of industrial contaminant isolates incorrectly identified as *Burkholderia lata*

**DOI:** 10.1099/mgen.0.000564

**Published:** 2021-04-23

**Authors:** Edward Cunningham-Oakes, Tom Pointon, Barry Murphy, Stuart Campbell-Lee, Gordon Webster, Thomas R. Connor, Eshwar Mahenthiralingam

**Affiliations:** ^1^​ Cardiff University, Microbiomes, Microbes and Informatics Group, Organisms and Environment Division, School of Biosciences, Cardiff University, CF10 3AX, UK; ^2^​ Unilever Research and Development, Port Sunlight, Bebbington, CH63 3JW, UK; ^3^​ Quay Pharmaceuticals Ltd, Quay House, 28 Parkway, Deeside Industrial Park, Flintshire, CH5 2NS, UK

**Keywords:** phylogenomics, *Burkholderia*, industrial microbiology, contamination, classification, taxon K

## Abstract

The *Burkholderia cepacia* complex (Bcc) is a closely related group of bacteria, composed of at least 20 different species, the accurate identification of which is essential in the context of infectious diseases. In industry, they can contaminate non-food products, including home and personal care products and cosmetics. The Bcc are problematic contaminants due to their ubiquitous presence and intrinsic antimicrobial resistance, which enables them to occasionally overcome preservation systems in non-sterile products. *Burkholderia lata* and *Burkholderia contaminans* are amongst the Bcc bacteria encountered most frequently as industrial contaminants, but their identification is not straightforward. Both species were historically established as a part of a group known collectively as taxon K, based upon analysis of the *recA* gene and multilocus sequence typing (MLST). Here, we deploy a straightforward genomics-based workflow for accurate Bcc classification using average nucleotide identity (ANI) and core-gene analysis. The workflow was used to examine a panel of 23 *Burkholderia* taxon K industrial strains, which, based on MLST, comprised 13 *B. lata,* 4 *B. contaminans* and 6 unclassified Bcc strains. Our genomic identification showed that the *B. contaminans* strains retained their classification, whilst the remaining strains were reclassified as *Burkholderia aenigmatica* sp. nov. Incorrect taxonomic identification of industrial contaminants is a problematic issue. Application and testing of our genomic workflow allowed the correct classification of 23 Bcc industrial strains, and also indicated that *B. aenigmatica* sp. nov. may have greater importance than *B. lata* as a contaminant species. Our study illustrates how the non-food manufacturing industry can harness whole-genome sequencing to better understand antimicrobial-resistant bacteria affecting their products.

## Data Summary

The authors confirm that all supporting data, code and protocols have been provided within the article. Illumina raw sequence reads and associated genomes have been deposited in the European Nucleotide Archive (ENA) under ENA project accession number PRJEB42964, with the exception of BCC1315, which was previously submitted under accession number ERA2359236.

**Impact Statement**

Industrial microbiology is an area where microbial misidentification is problematic, and the application of genomics is currently limited. To resolve the identification of problematic *Burkholderia cepacia* complex (Bcc) non-food product industrial contaminants, the genomes of 23 strains were sequenced and a genomic classification workflow was developed. We provide evidence to support the reclassification of misidentified industrial Bcc bacteria as the recently assigned *B. aenigmatica* sp. nov. The study provides an insight into how genomics may be utilized to improve the identification of Bcc bacteria, for which identification has historically relied upon the differential analysis of one (*recA*) or several (multilocus sequence typing) housekeeping genes. The findings of this study are beneficial to industry and medicine alike, due to the inherent difficulty in accurately identifying Bcc species, and the prevalent nature of named species *Burkholderia contaminans* and *Burkholderia lata* as cystic fibrosis pathogens and problematic contaminants of industrial products.

## Introduction

The *Burkholderia cepacia* complex (Bcc) is a group of closely related Gram-negative bacteria with a continuously evolving taxonomy, and currently comprises 20 named species [1, 2]. They have a diverse range of importance, including causing infection in people with cystic fibrosis (CF) [3] and plant pathogenesis [4], and, in relation to biotechnology, having roles in bioremediation and biological control [5] and being problematic industrial contaminants [6, 7]. Members of the Bcc are difficult to identify using traditional phenotypic microbiological techniques, as exemplified by their initial classification as *Pseudomonas cepacia* up until their taxonomic reclassification in 1997 [8]. Bcc bacteria often require selective media for their initial isolation [9], followed by molecular techniques such as 16S rRNA and *recA* gene sequencing [10], and multilocus sequence typing (MLST) [11] for accurate identification. Despite the widespread application of these molecular marker sequencing techniques, multiple novel species groups exist within the Bcc [1, 12].

MLST schemes have been developed for a multitude of bacteria of interest, including the Bcc [13]. MLST was designed to enable the unambiguous epidemiological characterization of closely related bacteria based on polymorphisms at seven conserved gene loci, from which bacteria are assigned a sequence type (ST) [14]. MLST analysis has previously identified the predominant STs amongst the Bcc in the context of CF infections, particularly global surveillance of the dissemination of ST types amongst CF cohorts [15, 16]. Moreover, MLST is a powerful tool for industry, where *Burkholderia* pose a contamination risk due their intrinsic antimicrobial and biocide resistance [17]. For example, *Burkholderia* isolates caused 3 % of European recalls due to microbial contamination of non-sterile non-food products between 2005 and 2018 [6]. Industrial microbiology and its associated identification standards are primarily based on low-resolution identification methods. Such techniques include culture enrichment and biochemical profiling [18], rapid but non-specific approaches such as ATP production assessment [19], or molecular techniques for general microbial identification, such as 16S rRNA gene sequencing [20]. These techniques are insufficient for accurate identification of certain Bcc species. The accurate characterization of bacteria of interest is a cornerstone of epidemiological investigations to ensure effective tracking, typing and control. This issue is exemplified by the proportion of microbial contaminants reported as unidentified in international databases for non-food product recalls, at 49 % of incidents [6]. However, with increased affordability and application of whole-genome sequencing [21], industry may now readily access the higher level of resolution afforded by genomics. In the case of the Bcc species, this has resulted in the discovery of novel species groupings, such as within *Burkholderia cenocepacia* [22], where genomics reclassified isolates identified as *B. cenocepacia* III-B by *recA* and MLST analysis [23, 24] as the proposed novel species *Burkholderia servocepacia* [22].

Taxon K (also known as group K) is a multi-species grouping within the Bcc that was proposed in 2009 based on analysis of the *recA* gene and MLST, and within which two novel species were formally named as *Burkholderia contaminans* and *Burkholderia lata* [25]. Subsequently, in 2013, *B. lata* was identified as one of the predominant Bcc species found as an industrial contaminant [26], and the model environmental *B. lata* type strain 383^T^ was shown to be capable of adapting its tolerance to a range of in-use preservatives, such as the isothiazolinones [26]. In a 2020 taxonomic study, Depoorter *et al*. [27] observed that Bcc taxon K bacteria split into at least three main species clades, encompassing the originally named species *B. lata* and *B. contaminans *, but also revealing *Burkholderia aenigmatica* sp. nov. as a new taxon with sufficient genetic and phenotypic differences to support its proposal as a new species. Going forward, the accurate identification of this preservative-tolerant species group in the industrial environment is crucial. In relation to Bcc industrial contaminants, the performance of existing molecular techniques such as *recA* and MLST that had been used to identify them [26] needs to be re-evaluated, particularly in light of further novel species being proposed in the taxon K group [27].

To date, few studies of non-food product microbial contaminants have made use of genomics to determine the species identity of problematic contaminants [6]. Herein, we use genomics to provide extensive taxonomic characterization of a panel of 23 Bcc isolates recovered from industrial contamination incidents, and previously identified as taxon K by MLST analysis. We show that the resolution provided by genomic taxonomy techniques accurately reclassifies industrial taxon K isolates within novel or existing species groups compared to MLST. Finally, we provide a straightforward genomics workflow for bacterial taxonomic identification in the context of industrial microbiology, which expands on past criteria using 16S rRNA gene and functional gene sequence analysis [28], and incorporates average nucleotide identity (ANI) analysis for greater resolution [29]. Application of this workflow to Bcc bacteria and taxon K species found as industrial contaminants provided an accurate and simple means of identification of these problematic micro-organisms.

## Methods

### DNA extraction and genome sequencing

Isolates were revived via plating onto tryptic soya agar, followed by inoculation of 3 ml tryptic soya broth for overnight culture, and were pelleted before genomic DNA was extracted. DNA extraction was performed on a panel of 23 historical contaminant isolates stored in the BCC collection at Cardiff University, using a Maxwell 16 Tissue DNA Purification kit and instrument (Promega, UK). These isolates were identified as a part of taxon K (Table 1) by MLST [26]. Within this panel, of 13 isolates that had previously been identified as *B. lata *, 4 were classified as *B. contaminans* and 6 were identified as a novel species subgroup within taxon K [26]. After Nextera XT (Illumina, UK) DNA library preparation, paired-end sequencing (150 bp) was performed on extracted genomic DNA using an Illumina NextSeq 500 platform at the Cardiff School of Biosciences Genomics Research Hub.

**Table 1.** Genome-sequenced industrial *Burkholderia cepacia* complex taxon K isolates used for identification workflow. Genome-derived sequence types (STs) highlighted in bold are known to comprise *B. aenigmatica* sp. nov, based on the findings of Depoorter *et al.* [27]. The type strain *B. aenigmatica* LMG 13014^T^ (accession: SAMEA5795692) was used for ANI comparison to the species-level identity. Historical IDs were obtained from the Cardiff Bcc collection database. ‘ANIm vs MLST type strain’ denotes the ANIm value obtained when comparing to the type strain of the species proposed by historical MLST ID

Strain	Genome size	Coverage	Assembly accession	Historical ID	Current MLST-assigned taxon*	Historical ST	Genome-derived ST	ANI (%) vs MLST type strain	ANI (%) vs * B. aenigmatica *	ANI ID
BCC1282	7.32	9.1	SAMEA7997990	Bcc novel taxon K	* B. aenigmatica *	333	333	na	99.9	* B. aenigmatica *
BCC1284	8.37	11.0	SAMEA7997991	* B. lata *	* B. aenigmatica *	98	**98**	94.6	95.7	* B. aenigmatica *
BCC1285	8.49	12.2	SAMEA7997992	* B. lata *	* B. aenigmatica *	98	**98**	94.7	95.8	* B. aenigmatica *
BCC1288	6.31	6.1	SAMEA7997993	* B. lata *	* B. aenigmatica *	98	**98**	94.8	95.7	* B. aenigmatica *
BCC1289	7.34	29.9	SAMEA7997994	* B. lata *	* B. aenigmatica *	98	**98**	94.7	95.7	* B. aenigmatica *
BCC1290	8.26	12.2	SAMEA7997995	* B. lata *	* B. aenigmatica *	98	**98**	94.8	95.8	* B. aenigmatica *
BCC1294	8.77	12.0	SAMEA7997996	* B. lata *	* B. aenigmatica *	98	**98**	94.7	95.8	* B. aenigmatica *
BCC1296	7.55	10.4	SAMEA7997997	* B. lata *	* B. aenigmatica *	119	**119**	94.7	95.8	* B. aenigmatica *
BCC1297	7.94	8.4	SAMEA7997998	* B. lata *	* B. aenigmatica *	119	**119**	94.8	95.8	* B. aenigmatica *
BCC1298	6.47	12.2	SAMEA7997999	* B. lata *	* B. aenigmatica *	119	**119**	94.8	95.8	* B. aenigmatica *
BCC1299	5.69	19.9	SAMEA7998000	* B. lata *	* B. aenigmatica *	119	**119**	94.8	95.7	* B. aenigmatica *
BCC1300	5.74	6.8	SAMEA7998001	Bcc novel taxon K	* B. aenigmatica *	334	334	na	97.6	* B. aenigmatica *
BCC1302	6.23	13.0	SAMEA7998002	Bcc novel taxon K	* B. aenigmatica *	333	**333**	na	99.9	* B. aenigmatica *
BCC1303	6.12	12.6	SAMEA7998003	Bcc novel taxon K	* B. aenigmatica *	333	**333**	na	99.9	* B. aenigmatica *
BCC1313**†**	6.51	12.1	SAMEA7998004	Bcc novel taxon K	na	335	335	na	98.8	* B. aenigmatica *
BCC1314**†**	5.13	17.5	SAMEA7998005	Bcc novel taxon K	na	336	336	na	97.5	* B. aenigmatica *
BCC1315	8.01	37.4	SAMEA6503210	* B contaminans *	* B. contaminans *	943	943	na	94.1	* B. contaminans *
BCC1321	4.43	17.2	SAMEA7998006	* B. lata *	* B. aenigmatica *	339	339	94.9	95.8	* B. aenigmatica *
BCC1323**†**	4.71	8.5	SAMEA7998007	* B. contaminans *	na	323	323	97.4	94.4	* B. contaminans *
BCC1406	4.29	5.8	SAMEA7998008	* B. lata *	* B. aenigmatica *	103	**103**	94.7	96.0	* B. aenigmatica *
BCC1554	7.31	25.3	SAMEA7998009	* B. lata *	* B. aenigmatica *	119	119	94.7	95.8	* B. aenigmatica *
BCC1582‡	6.36	16.5	SAMEA7998010	* B. contaminans *	na	na	102	99.9	94.5	* B. contaminans *
BCC1595‡	5.86	20.0	SAMEA7998011	* B. contaminans *	na	na	482	98.6	94.3	* B. contaminans *

*As reported by Depoorter *et al.* [27].

†ST does not have a species assigned within pubMLST.

‡Did not have a sequence type assigned prior to this study.

### Genome assembly, and development of a taxon K database for genomic analyses

Illumina adaptors were trimmed from 150-nucleotide paired-end reads using the TrimGalore script, and genomes were assembled using SPAdes v3.14.0 [30] and/or Unicycler v0.4.8 [31]. Assembled contigs were then screened for contamination using Kraken2 v2.0.8 [32]. A taxon K database was developed using 58 genomes, including the genomes sequenced in this study, and all available genomes in the ENA database for taxon K and related species, obtained using enaBrowserTools v1.5.4 (available via GitHub, https://github.com/enasequence/enaBrowserTools). This database included the complete genomes for type strains of *B. contaminans* (LMG 6992^T^), *B. lata* (383^T^), and the newly sequenced type strain of *B. aenigmatica* (LMG 13014^T^) [27].

### Determining the species-level identity of taxon K isolates

ANI analysis was conducted upon the taxon K genomes using the Python script PyANI v0.2.7 (available via GitHub, https://github.com/widdowquinn/pyani), using both blastn (generating an ANIb value) and MUMmer (generating an ANIm value) for alignment [31]. As defined previously, ANIb refers to pairwise ANI values calculated using the blastn algorithm, whilst ANIm refers to pairwise ANI results calculated with the MUMmer algorithm. Heatmaps were also generated using PyANI to provide a visualization of the level of identity between taxon K genomes. An ANI threshold of 95 % was used for analyses [29, 33], and genomes with an ANI value >95 % in comparison to a taxonomic type strain were designated as matching that species.

MLST profiles were generated using MLSTcheck [34] (available via GitHub: https://github.com/sanger-pathogens/mlst_check), which uses blastn to compare a query genome to PubMLST databases. All 58 genomes in the taxon K database were annotated using Prokka (v1.14.5) [35] under default parameters, before Roary (v3.12.0) [36] was used to generate a core-gene alignment of 255 genes, using the outputs of Prokka. The concatenated allele alignment produced by MLSTCheck was then used to generate a concatenated MLST allele phylogeny, whilst the 255 core-gene alignment produced by Roary was used to generate a phylogeny using RAxML [37]. A GAMMA model of rate heterogeneity supported by 100 bootstraps was used for both phylogenetic trees. Phylogenetic trees were then visualized using FigTree v1.4.2 [38] and edited using Inkscape v0.91 [39].

## Results

### ANI analysis confirms that the industrial isolates historically identified as *B. lata* or Bcc novel taxon K belong to *B. aenigmatica* sp. nov.

**Fig. 1.**
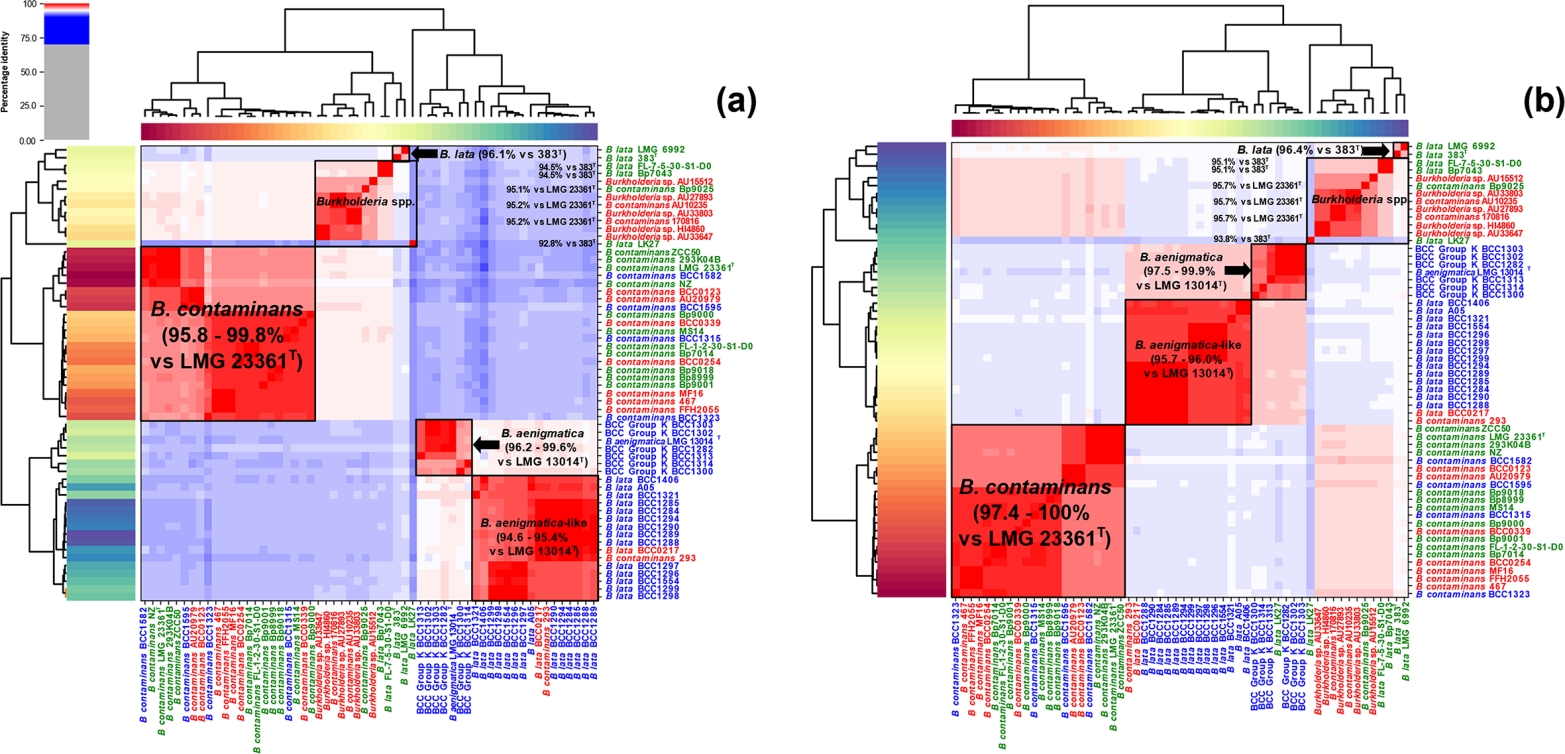
Taxonomic classification of taxon K isolates by average nucleotide identity. Heatmaps generated by PyANI using ANIb (**a**) and ANIm (**b**) for genome alignment are shown. The degree of genome similarity measured as percentage identity by means of pairwise comparison between genomes is indicated by the scale. Red areas highlight isolates that possess >95 % nucleotide similarity, with darker shades of red indicating greater similarity. Blue indicates <95% nucleotide similarity. Epidemiological information was obtained from European Nucleotide Archive metadata and used to highlight genomes as follows: strains highlighted in red are from a clinical background, strains highlighted in green are environmental and strains highlighted in blue are industrial.

**Fig. 2.**
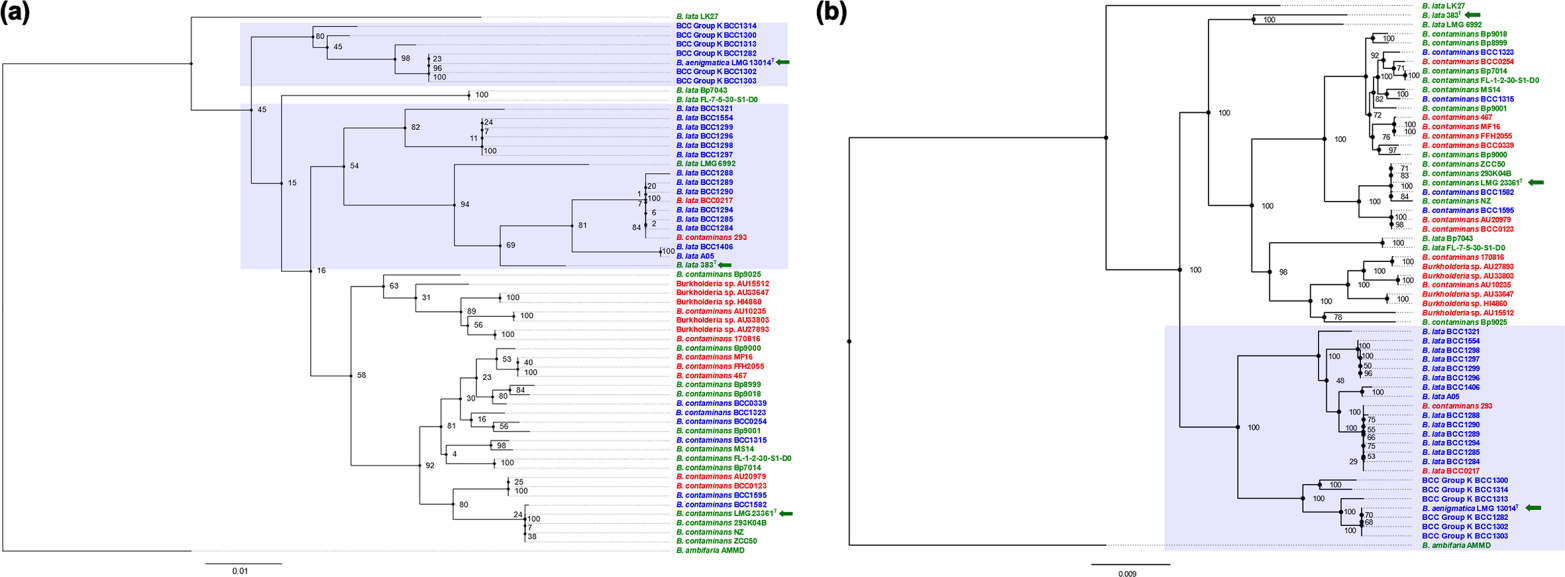
MLST and core-gene phylogenetic placement of *Burkholderia* taxon K industrial isolates. Both phylogenies were generated using RAxML with 100 bootstraps and were rooted with *Burkholderia ambifaria* AMMD. Scale bar represents the number of substitutions per base position. Type strains for *Burkholderia lata* (383^T^), *Burkholderia contaminans* (LMG 23361^T^) and *Burkholderia aenigmatica* (LMG 13014^T^) are denoted by green arrows. Shaded boxes indicate the phylogenetic position of *B. aenigmatica* isolates, as designated by average nucleotide identity (see Fig. 1). Epidemiological information was obtained from European Nucleotide Archive metadata and used to illustrate strain background as follows: strains highlighted in red are from a clinical background, strains highlighted in green are environmental and strains highlighted in blue are industrial.

Of the 23 industrial isolates subjected to genomic analysis, ANI-based species-level identification (Fig. 1) was only concordant with the genomes previously classified as *B. contaminans* by MLST (Fig. 2a). All four industrial isolates displayed ANI values above the 95.0 % threshold [33] for species identification (ANIm=97.4–99.9 %) when compared to the *B. contaminans* LMG 23361^T^ species reference (Fig. 1). In contrast, all 13 industrial isolates identified as *B. lata* by MLST displayed ANI values below the threshold for species delineation (ANIm=94.6–94.9 %, see Table S2, available in the online version of this article) in comparison to the *B. lata* 383^T^ species reference. Interestingly, the most closely related complete genome by ANI was that of the *B. lata* A05, a strain of industrial origin, isolated from a mouthwash containing 0.2 % of the preservative chlorhexidine [40]. Comparing strain A05 to *B. lata* 383^T^ gave an ANIm of 94.6 % (Table S2), but strain A05 displayed ANIm values of 98.6–99.7 % (Table S2) in comparison to the 13 industrial isolates, suggesting that it had been incorrectly designated as *B. lata *. ANI also revealed the existence of several other incorrectly classified/species borderline taxon K genomes, originating from non-industrial sources and labelled as *B. contaminans* or *B. lata* within the database (see Fig. 1, Table S1).

Recently, the novel Bcc species *B. aenigmatica* has been proposed after phenotypic and genomic examination of taxon K isolates [27]. Comparison to the *B. aenigmatica* type strain LMG 13014^T^ revealed that the six unidentified taxon K industrial isolates belonged to *B. aenigmatica* (ANIm=97.5–99.9 %, Table S2). This was also supported by the MLST sequence types matching those of known *B. aenigmatica* types (Table 1; see below). The remaining 13 isolates previously identified as *B. lata* formed a *B. aenigmatica *-like species grouping (ANIm=95.7–96.0 %, Table S2). Using a 95 % ANI threshold, these isolates were designated as *B. aenigmatica *. It is important, however, to note that that this ANI value is set arbitrarily based upon previous analyses of prokaryotic genomes [33], and that ambiguity exists around this threshold. As such, true genomic delineation should be achieved using values derived from the context of a given dataset (e.g. 97 % for taxon K, see the Discussion).

### Comparison of MLST and core-gene phylogenomics clarifies past misidentification of taxon K isolates and identifies *B. aenigmatica* as a problematic contaminant

MLST analysis based on *in silico* gene extraction from the genomes of all industrial isolates corroborated their historical ST identification (Table 1). Phylogenetic analysis of the concatenated MLST alleles placed the four *B. contaminans* industrial isolates [26] alongside the *B. contaminans* type strain LMG 23361^T^ (Fig. 2a). This MLST analysis also placed the 13 industrial isolates previously identified as *B. lata* [26], the *B. aenigmatica* [27] type strain, and the additional *B. lata* industrial isolate A05 [40], as adjacent to the *B. lata* species type strain 383^T^ (Fig. 2a).

To improve the phylogenomic resolution, core-gene analysis was used to generate an alignment of 255 core-genes for all taxon K genomes examined (Fig. 2b). The core-gene alignments provided an altered phylogenetic topological definition that contrasted with the MLST phylogeny but corroborated ANI analysis (Fig. 1a, b). The five *B. contaminans* isolates still placed alongside the type strain in this analysis. All 13 industrial isolates defined as *B. lata* by MLST (Fig. 1a) were clustered within a phylogenetically distinct group (shaded box, Fig. 2b), which did not include the type strain *B. lata* 383^T^. A number of isolates that clustered within this clade possessed STs (ST98, ST119, and ST333, Table 1) that are now recognized as belonging to *B. aenigmatica* sp. nov [27]. Moreover, a distinct phylogenetic split exists within this clade, with *B. aenigmatica* LMG 13014^T^ forming a subclade with the six previously unclassified taxon K isolates, and the remaining isolates forming a closely related subclade, with *B. lata* A05 being the most phylogenetically similar complete genome. This split is supported by both ANIb (Fig. 1a) and ANIm (Fig. 1b), where *B. aenigmatica* and *B. aenigmatica *-like isolates form two distinct alignment clusters. Overall, core-gene phylogenetic analysis was concordant with both ANIm and ANIb, and the designation of 6 taxon K isolates and 13 taxon K isolates [26] as *B. aenigmatica *. This dataset also revealed a unique intraspecies sub-clading, for which a limited amount of evidence was observed in previous datasets [27].

## Discussion

**Fig. 3.**
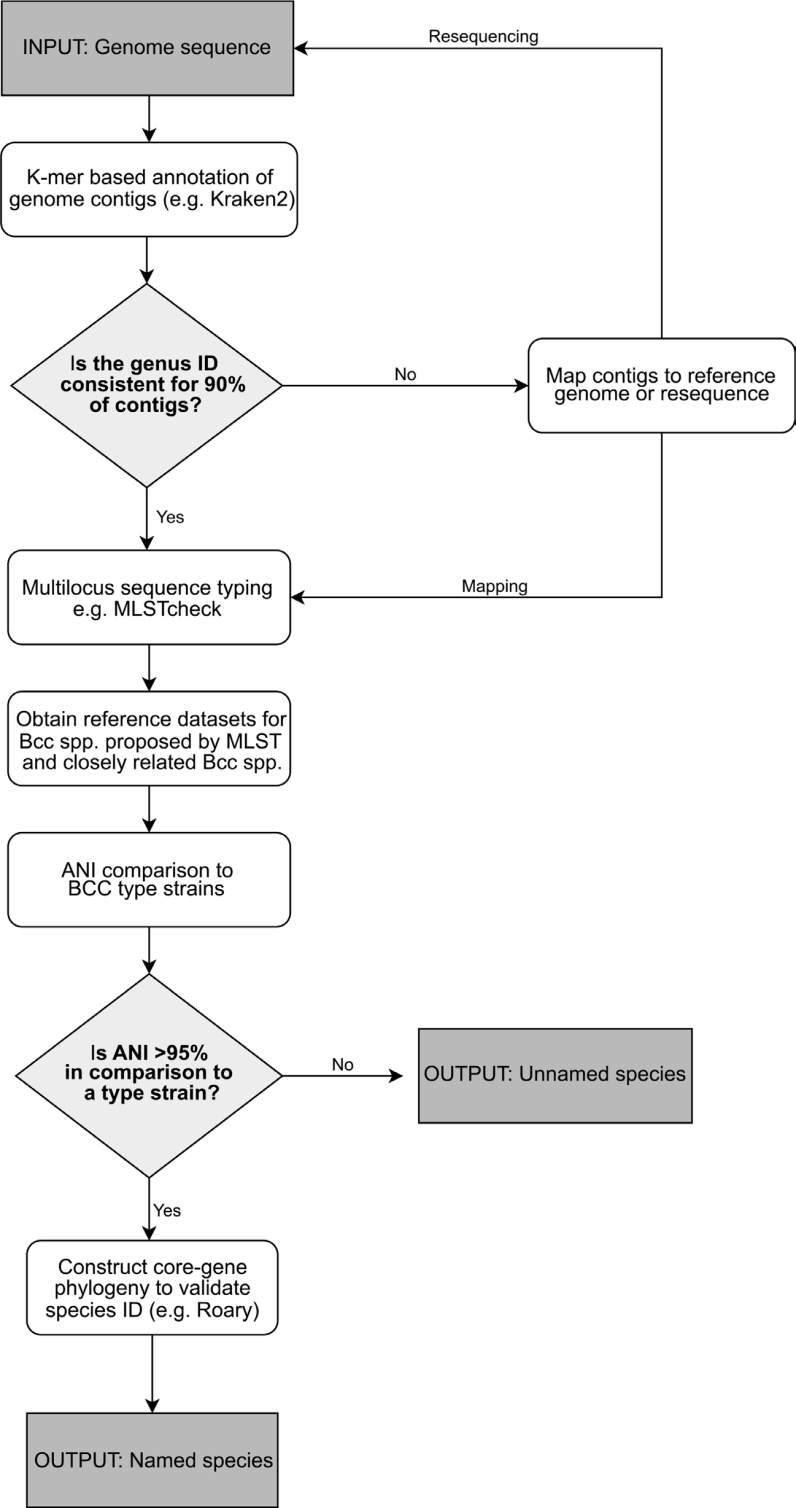
Proposed workflow for the identification of *Burkholderia cepacia* complex species. The analysis workflow is dependent upon an initial identification by multilocus sequence typing in order to provide a preliminary species-level identification, before moving to identification using genome to genome comparison by means of average nucleotide identity and core-genome phylogenetic comparisons to confirm species nomenclature.

Overall, the data presented illustrate a clear workflow for the identification of Bcc species as important industrial contaminants and clinical bacteria. This genomic workflow expands on past minimum taxonomic criteria [26] and presents a straightforward series of analyses that can accurately classify taxonomically complex bacteria such as Bcc species (Fig. 3). It supports the view that although MLST, as one of the most widely applied methods in relation to identification of Bcc bacteria, can be used to generate a preliminary profile of isolates of interest and group them into the Bcc, further genomic analysis is required to accurately classify isolates within certain groups such as taxon K. MLST was not able to accurately identify the taxon K industrial isolates correctly to the species level (Fig. 2a). This is evidenced by the core-gene phylogenetic analysis and ANI, placing 19 taxon K isolates previously identified as *B. lata* or as unidentified by MLST into *B. aenigmatica* sp. nov. This distinction is not shown by blast or phylogenetic analysis of MLST genes, both of which place these isolates alongside the *B. lata t*ype strain, 383^T^, or do not provide species-level identitification at all.

Although a 95–96 % ANI threshold [33] is accepted as a primary delineator of bacterial species identity, and it could be argued that the 13 *B. aenigmatica *-like isolates belong the *B. aenigmatica* sp. nov., ANI thresholds should be chosen as appropriate for a given dataset, as it is also known that there is genetic discontinuity around this boundary [29]. In the context of this taxon K dataset, we chose 95 % as the threshold for ANI, thereby classifying all 19 isolates as *B. aenigmatica *. However, examining higher or lower ANI boundaries for different groups of bacteria may aid classification (Fig. 1). In the case of Bcc taxon K strains, we observed that a 97 % ANI threshold provided additional support to the separation of *B. lata *, *B. contaminans* and novel species isolates within the taxon K contaminant collection. This is clearly shown in our analyses, which demonstrate a strong concordance between core-gene phylogenetic clading (see Fig. 2b), and ANI (Fig. 1a, b), where ANI value ranges are shown for each species grouping) if a 97 % threshold was to be used.

To conclude, techniques that analyse differences throughout the genome, as opposed to between a limited number of conserved genes such as MLST, are essential to accurately identify Bcc species going forward. To this end, core-gene analysis [41] and ANI [29, 33] should be considered the gold standard techniques for the accurate identification of taxon K species. Furthermore, given that all of the *B. lata* industrial isolates sequenced in this study and the mislabelled deposited industrial strain A05 genome (Tables 1 and S1) are members of the new taxa *B. aenigmatica* [27], the identity of all industrial and clinical strains reported as *B. lata* should be re-evaluated. This is an important identification correction in relation to industrial microbiology, as from 2013 *B. lata* was observed to be the most common species present in a collection of 60 contaminant strains [26]. The fitness of *B. aenigmatica* sp. nov. in relation to preservative tolerance and survival within industrial products remains to be determined. However multiple members of the taxon K group are intrinsically resistant to antimicrobials and have been identified as problematic contaminants [6, 7, 26]. The elucidation of this novel taxonomic grouping *B. aenigmatica* playing a role in contamination, and the contribution of a large panel of industrial genomes to public databases, will be invaluable in the further development of the taxonomy of *Burkholderia* species.

## Supplementary Data

Supplementary material 1
